# Tumors of the parapharyngeal space: the VU University Medical Center experience over a 20-year period

**DOI:** 10.1007/s00405-018-4891-x

**Published:** 2018-02-07

**Authors:** Thijs van Hees, Stijn van Weert, Birgit Witte, C. René Leemans

**Affiliations:** 1 Department of Otolaryngology and Head and Neck Surgery, VU University Medical Center, De Boelelaan 1117, 1081 HV Amsterdam, The Netherlands; 2 Department of Epidemiology and Biostatistics, VU University Medical Center, Amsterdam, The Netherlands

**Keywords:** Parapharyngeal space, Diagnostics, Treatment, Outcome

## Abstract

**Background:**

Tumors of the parapharyngeal space (PPS) are rare, accounting for 0.5–1.5% of all head and neck tumors. The anatomy of the PPS is responsible for a wide variety of tumors arising from the PPS. This series of 99 PPS tumors provides an overview of the clinical course and management of PPS tumors.

**Materials and methods:**

This retrospective study included clinical data from patients treated for PPS tumors from 1991 to 2012 (warranting at least a 4-year follow-up) at the VU University Medical Center, Amsterdam, The Netherlands.

**Results:**

Fifty percent were salivary gland tumors, 41% were neurogenic and 9% had a different origin. 18.2% of the PPS tumors were malignant. The most reported symptom at presentation was swelling of the neck and throat. In 14%, the PPS tumor was an accidental finding following imaging for other diagnostic reasons. Cytology showed an accuracy rate of 73.1% (19/26). The positive predictive value of a malignant cytology result was 86% (95% CI 42.1–99.6%). Surgery was performed in 55 patients (56%). The most frequently performed approach (56%) was the cervical–transparotid approach, followed by the cervical (25%), transmandibular (16%) and transoral (2%) approach. Nine patients died of the disease, of which seven patients had a malignant salivary gland tumor, one patient had a pleomorphic adenoma at first diagnosis which degenerated into carcinoma ex pleomorphic adenoma and one patient died of metastatic renal cell carcinoma.

**Conclusion:**

This large single-centre report on PPS tumors shows that careful diagnostic work up and proper surgical planning are important in this specific and rare group of head and neck tumors. Surgery was the main treatment (56%) for parapharyngeal tumors. Management of parapharyngeal neurogenic neoplasms generally consists of active surveillance due to peri-operative risk for permanent cranial nerve damage. The histopathological diagnoses were consistent with previous reports.

## Introduction

The parapharyngeal space (PPS) is a triangular fat-filled compartment of the suprahyoid neck, lateral to the pharynx. It is described as an inverted pyramid with the floor of the pyramid at the skull base and the apex at the level of the greater cornu of the hyoid bone. There are several detailed anatomic descriptions in the literature [1–5]. The styloid process along with the attaching muscles and tensor veli palatini fascia divides the PPS into a prestyloid and poststyloid compartment. The prestyloid space contains the deep parotid lobe and minor salivary glands. The poststyloid space contains the IX, X, XI and XII cranial nerves, the cervical sympathetic chain, the internal jugular vein, the internal carotid artery and lymph nodes. This typical anatomy of the PPS is responsible for a wide variety of tumors arising from the PPS [1, 6].

Tumors of the PPS account for 0.5–1.5% of all head and neck tumors [2, 7]. The majority is diagnosed in adults and include primary neoplasms, direct extension from adjacent regions and metastatic disease. Approximately, 80% of these neoplasms are benign [2, 5, 7–11]. Benign salivary gland tumors, and specifically pleomorphic adenoma, are most common, followed by neurogenic tumors [5, 10–12]. Salivary gland neoplasms are also the most frequently found primary malignant tumors [3, 5, 7, 10]. Prestyloid tumors are commonly from salivary origin, while the poststyloid space is most often affected by neurogenic tumors.

PPS tumors may stay undetected for a long time because they initially develop without causing any symptoms. Symptoms generally occur when the tumor becomes larger than 2.5–3 cm [8, 10]. Imaging is crucial for the assessment of PPS tumors. Magnetic resonance (MR), computed tomography (CT) with contrast and contrast angiography, in selected cases, are essential for diagnosis. MR is preferred over CT because it provides more useful information on tumor localization and extension and distinguishes different tumor types as well as possible malignant characteristics and their relation to adjacent structures. Imaging also aids in preoperative planning [8].

The cervical and the cervical—transparotid approach are generally used for surgical access. Under certain circumstances a cervical—transmandibular and combined transoral approach may be required [1, 7, 8]. The risk of tumor spill is more likely if the access is limited, the tumor is large or in case of underestimation of the tumor’s dimensions. Several supplementary procedures for a better access have been described, i.e., division of the stylomandibular ligament, removal or mobilization of the submandibular gland, excision of the lateral lobe of the parotid gland, extirpation of the styloid process and division of the posterior belly of the digastric muscle from the hyoid. Identification of the facial nerve and removal of the parotid tissue around the site of the origin will help to avoid tumor spill [1].

We examined the clinical data of 99 patients treated at the VU University Medical Center for PPS tumors. This study is a follow-up of the study of Allison (1989) in this department [9].

## Materials and methods

This retrospective study included patients treated for PPS tumors from 1991 to 2012 at the department of Head and Neck Surgery of the VU University Medical Center, Amsterdam, the Netherlands. During this period, treatment strategies remained unchanged. All patients with tumors in the PPS were included. Tumors originating from the deep lobe of the parotid gland were only included when fully located in the PPS.

Medical records were reviewed. The data obtained were symptoms, clinical signs, diagnostic procedures, surgical approaches, complications and histopathological findings. The preoperative imaging (MR and CT) was reviewed. Parameters determined on MR and CT were; tumor size, localization in the pre- or poststyloid space and tumor characteristics.

The surgical approach was dictated by the localization of the tumor, its dimensions, relation to anatomic structures and its etiology. Follow-up was carried out on a regular basis with clinical examination and MR or CT of the head and neck in case of proven malignancy and/or peri-operative spill. Management of parapharyngeal neurogenic neoplasms mostly consists of active surveillance due to peri-operative risk for permanent cranial nerve damage.

Data obtained were statistically processed with SPSS statistics 22 (IBM, Chicago, USA). The Fisher’s exact test and exact confidence intervals were used.

## Results

Ninety-nine patients with a tumor of the PPS were treated over a 20-year period of whom 38 (38.4%) were male. The median age was 49 years (range 4–87 years) and the median follow-up time was 53 months. 18.2% of the PPS tumors were malignant which is in accordance with previous reports [2, 7–11]. The different tumor types are listed in Table 1. Fifty percent were salivary gland tumors, 41% were neurogenic and 9% had a different origin.

**Table 1:** Diagnoses

Salivary gland neoplasms	49.5%
Benign salivary gland neoplasms	36.4%
Pleomorphic adenoma	31 (31.3%)
Monomorphic	4 (4.0%)
Warthin	1 (1.0%)
Malignant salivary gland neoplasms	13.1%
Adenoid cystic carcinoma	4 (4.0%)
Carcinoma ex pleomorphic adenoma	3 (3.0%)
Adenocarcinoma NOS	3 (3.0%)
Acinic cell carcinoma	2 (2.0%)
Myoepithilial carcinoma	1 (1.0%)
Neurogenic neoplasms	41.4%
Paraganglioma	30 (30.3%)
Carotid body paraganglioma	20
Vagal paraganglioma	17
Jugular paraganglioma	1
Schwannoma	9 (9.1%)
Neurofibroma	2 (2.0%)
Miscellaneous lesions	9.1%
(Lympho)vascular malformation	2 (2.0%)
Lymphoma non-Hodgkin	1 (1.0%)
Rhabdomyoma	1 (1.0%)
Rhabdomyosarcoma	1 (1.0%)
Branchial cyst	1 (1.0%)
Metastasis	3 (3.0%)

Table 2 summarizes the main presenting symptoms and clinical signs. The most reported symptom at presentation was swelling of the neck and throat. Unlike other studies, we did not find any complaints of trismus in our group [2, 9, 11, 13, 14]. In 14 patients (14.1%), the PPS tumor was an accidental finding following imaging for other diagnostic reasons. All accidentally found parapharyngeal tumors were benign (*p* = 0.118); 69% salivary, 23% neurogenic and 8% of other origin.

**Table 2:** Incidence and frequencies of symptoms and clinical signs

Symptoms		Clinical signs	
Swelling neck	46 (46.5%)	Neck mass	57 (57.6%)
Intraoral mass	22 (22.2%)	Oropharyngeal mass	58 (58.6%)
Dysphagia	15 (15.2%)	Cranial nerve palsy	13 (13.1%)
Tube dysfunction	13 (13.1%)	Facial	3 (3.0%)
Dysphonia	10 (10.1%)	Glossopharyngeal	1 (1.0%)
Otalgia	9 (9.1%)	Vagal	8 (8.1%)
Lump in throat	9 (9.1%)	Accessory	1 (1.0%)
Sore throat	8 (8.1%)	Hypoglossal	5 (5.1%)
Dyscomfort	6 (6.1%)	Serous otitis media	10 (10.1%)
Hearing loss	5 (5.1%)		
Snoaring	5 (5.1%)		
Pulsatile tinnitus	5 (5.1%)		
Facial nerve paresis	3 (3.0%)		

During physical examination, swelling of the neck and the oropharynx was encountered most. Thirteen cases presented with cranial nerve palsy, mostly from the vagal nerve (see Table 2). Otitis media with effusion was diagnosed in 10% of cases.

MR, CT, angiography and cytology were the diagnostic methods used. MR and CT were applied in 94 and 25%, respectively, angiography was performed in 15%, which were mostly cases of paragangliomas. Cytology, mainly ultrasound-guided fine needle aspiration (USgFNAC), was done in 58%, paragangliomas precluded (30%). In 35% (*n* = 20) cytology was non-diagnostic due to inadequate cellular material, leaving 37 patients with a diagnostic cytology result. Of these 37 patients, 26 underwent surgery and cytology results could be confirmed with definitive histology. The overall cytology accuracy rate (73.1%) as well as the positive predictive value (PPV) as the negative predictive value (NPV) per definitive diagnosis are depicted in Table 3. 21.5% of non-diagnostic cytology revealed a malignant tumor after surgery, which is comparable with the 1 minus NPV of malignant cytology.

**Table 3:** Predictive value of cytology

Definitive diagnosis	Cytology PPV	Cytology NPV
Malignancy	85.7%	78.9%
Pleomorphic adenoma	76.4%	100.0%
Adenoid cystic carcinoma	100%	95.8%
Acinic cell carcinoma	50%	100%
Overall cytology accuracy rate	73.1%	

**Fig. 1:**
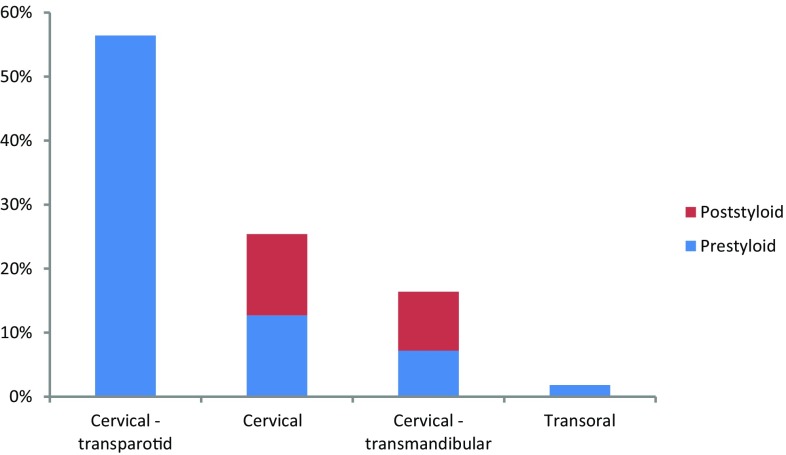
Used surgical approaches

**Fig. 2:**
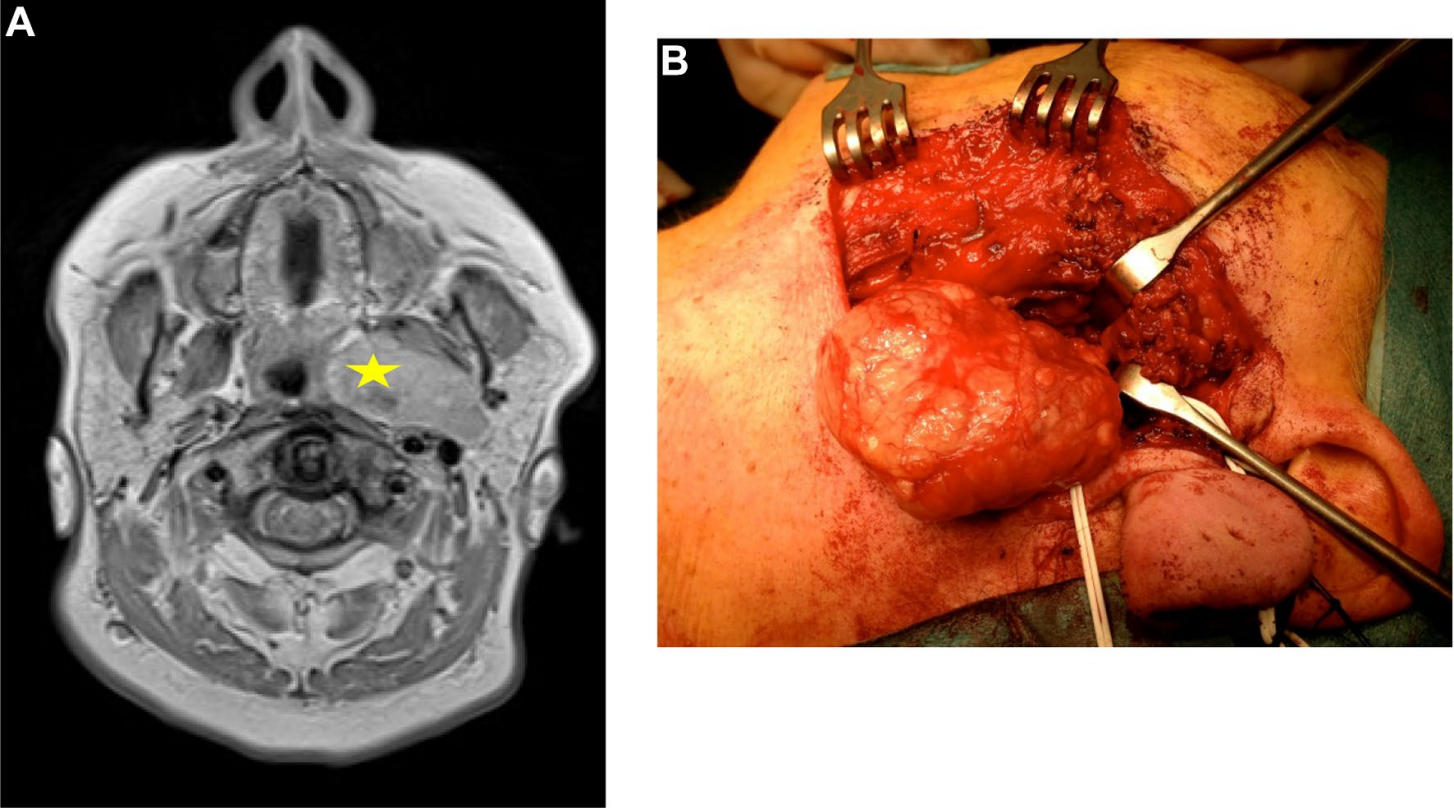
**a** Axial T2-weighted image of a pleomorphic adenoma (star) extending from the deep lobe of the left parotid. **b** Intra-operative image of removal through a cervical—transparotid approach. The stylomandibular ligament was cut to allow delivery of the tumor through the neck. The superficial lobe was repositioned after mobilization for access

Surgery was performed in 55 patients (56%). Thirty-eight patients, mainly diagnosed with paragangliomas, remained under active surveillance. Patients with malignant salivary tumors, pleomorphic adenomas and schwannomas were mainly treated surgically. Other primary treatment modalities used were radiotherapy (*n* = 3) and chemoradiation (*n* = 2). Post-operative radiotherapy (PORT) was given in 13 cases. An overview of the surgical approaches is shown in Fig. 1. The most frequently performed approach (56%) was the cervical—transparotid approach. An example of a pleomorphic adenoma extending from the deep lobe of the left parotid is shown in Fig. 2. In 12 (39%) patients, this approach was assisted by transoral dissection. In 25%, a cervical approach was applied, of which 43% was assisted transorally, and in 16% the cervical approach was extended with a mandibular osteotomy. A transoral approach only was performed once. The cervical—transparotid and the transoral approaches were only performed in cases with prestyloid tumors. The cervical approach was used for pre- and poststyloid tumors equally. The cervical—transmandibular approach was performed in 44% for cases of prestyloid tumors.

In 86% of cases, a macroscopic radical resection was achieved. In 10% macroscopic residual tumor was (deliberately) left behind (f.e. schwannoma of the vagus nerve). In 4% of cases, surgical reports were irretrievable. Short-term post-operative complications according to the Clavien–Dindo classification were facial nerve palsy, wound infection and hemorrhage. (Table 4). The most frequently encountered long-term post-operative complications were first-bite syndrome (*n* = 9; 16.4%) and Frey’s syndrome (*n* = 4; 7.3%). The median follow-up period was 53 months. In the group of benign tumors, three patients had a recurrent tumor, which were two carotid body paragangliomas and one pleomorphic adenoma. There were four recurrences in the group with malignant tumors; one adenocarcinoma, one acinic cell carcinoma (AciCC), one adenoid cystic carcinoma (ACC) and one myoepithelial carcinoma. The median time to develop a recurrence was 19 months. Tumor spill was not associated with recurrence in this study.

**Table 4:** Complications according to the Clavien–Dindo classification

Post-operative		
Grade I	Facial nerve palsy	12 (21.8%)
Grade II	Wound infection	6 (10.9%)
Grade III	Hemorrhage	2 (3.6%)

Seven patients (39%) died of their malignant parapharyngeal salivary tumor. One patient with a pleomorphic adenoma at first diagnosis, died of disease. Because of old age, active surveillance was preferred over surgical intervention. After 6 years, however, the tumor had degenerated into a carcinoma ex pleomorphic adenoma and the patient died of her disease. One patient with a metastasis of a renal cell carcinoma tumor died. None of the patients with a neurogenic tumor died of their disease.

## Discussion

PPS tumors are challenging to treat. The aim of this study was to evaluate the symptoms, clinical findings, diagnostic procedures, surgical approaches, histopathological findings and results of follow-up.

This study presents a series of 99 PPS tumors with a wide variety of diagnoses. The distribution of benign and malignant tumors was 4:1 and is in accordance with previous reports [2, 7, 9–11]. Salivary tumors were predominant for both benign and malignant tumors, followed by neurogenic tumors (40%), which is comparable with other studies [5, 7–13]. The prevalence of paragangliomas in the present study was 30% which is well within the range of previous reports (10–40%) [2, 5, 7, 10, 11, 13]. ACC was the most prevalent malignant tumor, followed by carcinoma ex pleomorphic adenoma and metastatic disease.

A PPS tumor generally presented as a swelling in the neck [15]. Other symptoms were reported (Table 2), including swelling of the throat, dysphagia and tuba dysfunction. Facial nerve palsy was rare (3%) and always associated with malignancy. This is in accordance with other studies. Ten percent was asymptomatic at time of presentation.

With respect to imaging, MR was preferred because it is superior in defining soft tissue abnormalities and providing important information on malignant signs such as local invasion, regional metastasis and perineural invasion. Adequate preoperative work up facilitates counselling of the patient, peri-operative planning and reduces the chance of tumor spill or irradical resection [16]. On MR, the tumors’ localisation is depicted in relation to the great vessels signifying pre- or poststyloid position. In 57 patients FNAC was performed. The accuracy rate was 73.1%. FNAC was obviously not performed in cases of suspicion of a vascular tumor such as a paraganglioma. Despite the relatively high PPV of cytology for malignancy (86%), clinical behaviour and radiographic aspects have to be taken into account. Management of parapharyngeal neurogenic neoplasms is mostly active surveillance due to peri-operative risk for permanent cranial nerve damage. Surgery was performed in 55 out of 99 patients (56%). Three main surgical approaches were used, guided by tumor size, histological type and position of the tumor in relation to the major vessels. The most frequently performed surgical approach was the cervical—transparotid approach (56%), which is used for prestyloid tumors arising from the deep lobe of the parotid gland. Effort is made to preserve the superficial lobe of the parotid gland to reduce the risk of developing Frey’s syndrome and for a better cosmesis. The cervical approach was employed (25%) for both pre- and poststyloid tumors. This approach provides direct access to the PPS. For optimizing exposure transection of the stylomandibular ligament, excision of the submandibular gland and/or styloid process is optional. The cervical approach can be transformed in a cervical—transparotid approach by extension of the incision.

The cervical—transmandibular approach was employed in 16%, slightly more than < 10% in other studies [8, 10, 11, 17–19]. The transmandibular approach was reserved for highly selected cases as recurrence of pleomorphic adenoma and very large tumors. The transoral approach was performed only once. The combination of a transoral incision and a cervical approach was employed if needed. Recently, Duek et al. reported on a case in which transcervical endoscopic and transoral robotic surgery was used [20]. With the increasing interest in robotic head and neck surgery, this might be an interesting adjunct to the known approaches.

Of the malignant cases (18), surgical excision was performed in 14 cases, of which 12 patients received PORT. The two cases without PORT harbored postoperatively proven metastatic disease. Four patients (rhabdomyosarcoma, non-hodgkin lymphoma, distant metastatic disease and inoperable ACC) received primary (chemo)radiotherapy.

Three benign and four malignant recurrences were registered. The median time of developing a local recurrence was 19 months. Seven patients—all surgically treated—died of a malignant parapharyngeal salivary gland tumor. One patient was first diagnosed with a pleomorphic adenoma, treated conservatively, and developed a carcinoma ex pleomorphic adenoma with perineural growth and died of her disease.

This large single-centre report on PPS tumors shows that careful diagnostic work up and proper surgical planning are important in this specific group of head and neck tumors. Management of PPS tumors is primarily defined by the tumor’s histology, followed by its localization (pre- or poststyloid) and size. One should keep in mind that FNAC is mandatory and helpful when feasible in diagnostic work up, but that discrepancy with definitive histology of the surgical specimen should not be underestimated. MR imaging specifically can narrow down the differential diagnosis due to its ability to distinguish malignant characteristics such as perineural involvement.

## References

[1] Bradley PJ, Bradley PT, Olsen K (2011). Update on the management of parapharyngeal tumours. Curr Opin Otolaryngol Head Neck Surg.

[2] Carrau R, Meyers E, Johnson J (1990). Management of tumors arising in the parapharyngeal space. Laryngoscope.

[3] Olsen K (1994). Tumors and surgery of the parapharyngeal space. Laryngoscope.

[4] Stambuk H, Patel S (2008). Imaging of the parapharyngeal space. Otolaryngol Clin N Am.

[5] Batsakis J, Sneige N (1989). Parapharyngeal and retropharyngeal space diseases. Ann Otol Rhinol Laryngol.

[6] Starek I, Mihal V, Novak Z, Pospisilova D, Vomacka J, Vokurka J (2004). Pediatric tumors of the parapharyngeal space. Three case reports and a literature review. Int J Pediatr Otorhinolaryngol.

[7] Shabab R, Heliwell T, Jones A (2005). How we do it: a series of 114 primary pharyngeal space neoplasms. Clin Otolaryngol.

[8] Dimitrijevic M, Jesic S, Mikic A, Arsovic N, Tomanovic N (2010). Parapharyngeal space tumors; 61 case reviews. Int J Oral Maxillofac Surg.

[9] Allison R, Van der Waal I, Snow G (1989). Parapharyngeal tumours: a review of 23 cases. Clin Otolaryngol.

[10] Zhi K, Ren W, Zhou H, Wen Y, Zhang Y (2009). Management of parapharyngeal-space tumors. J Oral Maxillofac Surg.

[11] Riffat F, Dwivedi RC, Palme C, Fish B, Jani P (2014). A systematic review of parapharyngeal space tumors reported over 20 years. Oral Oncol.

[12] Mendelsohn A, Bhuta S, Calcaterra T, Shih H, Abemayor E (2009). St. John M. parapharyngeal space pleomorphic adenoma: a 30-year review. Laryngoscope.

[13] Hughes K, Olsen K, McCaffrey T (1995). Parapharyngeal space neoplasms. Head Neck.

[14] Pensak M, Gluckman J, Shumrick K (1994). Parapharyngeal space tumors: an algorithm for evaluation and management. Laryngoscope.

[15] Ijichi K, Murakami S (2017). Surgical treatment of parapharyngeal space tumors: a report of 29 cases. Oncol Lett.

[16] Sun F, Yan Y, Wei D, Li W, Cao S, Liu D, Li G, Pan X, Lei D (2018). Surgical management of primary parapharyngeal space tumors in 103 patients at a single institution. Acta Otolaryngol.

[17] Chijiwa H, Mihiki T, Shin B, Sakamoto K, Umeno H, Nakashima T (2009). Clinical study of parapharyngeal space tumours. J Laryngol Otol.

[18] Pang K, Goh C, Tan H (2002). Parapharyngeal space tumours: an 18 year review. J Laryngol Otol.

[19] Cohen S, Burkey B, Netterville J (2005). Surgical management of parapharyngeal space masses. Head Neck.

[20] Duek I, Amit M, Sviri GE, Gil Z (2017). Combined endoscopic transcervical-transoral robotic approach for resection of parapharyngeal space tumors. Head Neck.

